# Supplementary material for: CRISPR/Cas9 bioluminescence-based assay for monitoring CFTR trafficking to the plasma membrane
Source: Life Sci Alliance. 2023 Nov 2;7(1):e202302045. doi: 10.26508/lsa.202302045 (PMC10622324; doi:10.26508/lsa.202302045)
Supplement: Supplementary file 3 [file LSA-2023-02045_SdataF5.pdf]

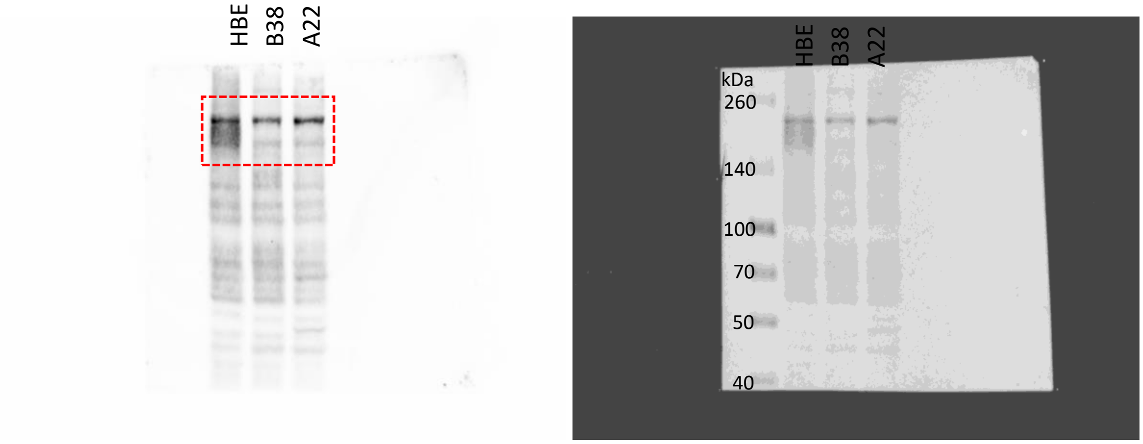

Western Blotting CFTR 596 Ab: 16HBE14o- parental (HBE); B38 clone; A22 clone

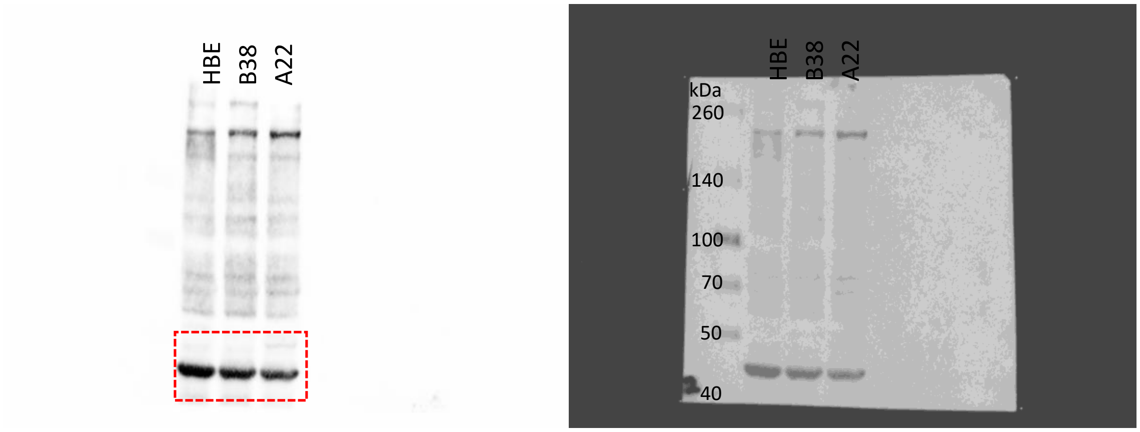

Western Blotting β-actin Ab: 16HBE14o- parental (HBE); B38 clone; A22 clone

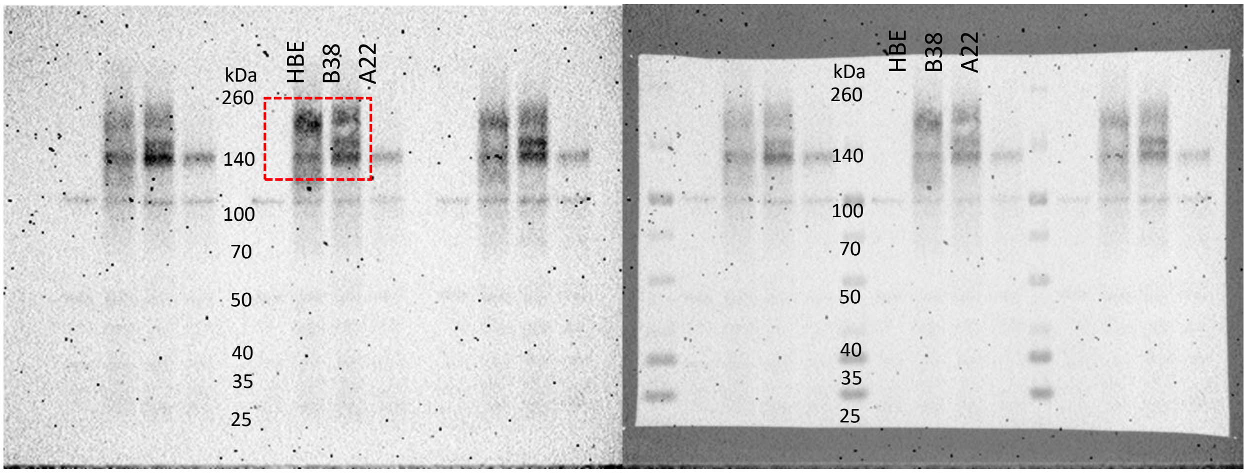

HiBiT Blotting (HiBiT tag detection): 16HBE14o- parental (HBE); B38 clone; A22 clone
